# Rapamycin Attenuates High Glucose-Induced Inflammation Through Modulation of mTOR/NF-κB Pathways in Macrophages

**DOI:** 10.3389/fphar.2019.01292

**Published:** 2019-10-30

**Authors:** Jiezhi Dai, Chaoyin Jiang, Hua Chen, Yimin Chai

**Affiliations:** Department of Orthopedic Surgery, Shanghai Jiao Tong University, Affiliated Sixth People’s Hospital, Shanghai, China

**Keywords:** diabetic wound, NLRP3 inflammasome, mTOR, NF-κB, Rapamycin

## Abstract

**Background:** The NLRP3 inflammasome is one of the key contributors to impaired wound healing in diabetes. In this study, we assessed the role of rapamycin on high glucose-induced inflammation in THP-1-derived macrophages and investigated the underlying signaling mechanisms.

**Methods:** THP-1-derived macrophages were treated with high glucose to induce NLRP3 inflammasome activation. The cells were pretreated with rapamycin, BAY 11-7082, or PDTC before exposure to HG. mTOR, NF-κB, and NLRP3 inflammasome expression were measured by western blotting.

**Results:** We found that rapamycin reduced NLRP3 inflammasome activation in macrophages. Rapamycin reduced NLRP3 inflammasome activation by inhibiting mTOR phosphorylation and NF-κB activation. Moreover, mTOR siRNA inhibited NF-κB activation, leading to the suppression of NLRP3 inflammasome activation.

**Conclusion:** Rapamycin can ameliorate high glucose-induced NLRP3 inflammasome activation by attenuating the mTOR/NF-κB signaling pathway in macrophages. Rapamycin may act as a possible therapeutic option for high glucose-induced inflammatory response in impaired wound healing in the future.

Diabetes is a public health problem of considerable magnitude. It has been reported that up to 15% of all diabetics individuals are prone to developing foot ulcers during their lifetime (B. Deni Raja et al., 2017). The development of foot ulcers is associated with increasing morbidity and mortality and is the most common cause of lower extremity amputations (Ertugrul et al., 2012). Thus, further investigations of the pathogenesis of diabetic wounds and the improvements made in diabetic wound management are indispensable.

Recently, many studies have focused on the role of the NLRP3 inflammasome in metabolic disorders (Vandanmagsar et al., 2011). The NLRP3 inflammasome is a multiprotein complex consisting of the NLRP3, apoptosis-associated speck-like protein with CARD domain and pro-caspase-1 (Martinon et al., 2002). The inflammasome is a molecular platform for immune defense. Induction of the NLRP3 inflammasome triggers caspase-1 activation and subsequently leads to the secretion of IL-1β and IL-18 (Stefanie and Sutterwala, 2013). A previous study has indicated that persistent activation of the inflammasome in macrophages contributes to impaired wound healing in diabetes (Mirza et al., 2014). Our study also found increased levels of IL-1β in wounds from diabetic patients and rats, resulting in a persistent inflammatory response and impaired healing (Dai et al., 2017b; Zhang et al., 2017).

The mammalian target of rapamycin (mTOR) is a serine/threonine kinase (Huang et al., 2015). mTOR acts as a central regulator of immune responses and is involved in the differential regulation of pro- and anti-inflammatory cytokine levels (Temiz-Resitoglu et al., 2017). In the clinic, targeting the mTOR signaling pathway has been implicated as a potential treatment option for inflammation-related diseases (Madonna et al., 2015). As an inhibitor of mTOR, rapamycin can suppress nuclear factor kappa B (NF-κB) activity, and NF-κB promotes NLRP3 inflammasome activation resulting in pro-IL-1β secretion (Dhingra et al., 2013; Zhong et al., 2016). However, the effect of mTOR inhibition on high glucose-induced inflammation has not been well examined.

Given these multiple functions of rapamycin, in this study, we aimed to assess the roles of rapamycin in the regulation of high glucose-induced inflammation in THP-1-derived macrophages and to investigate the underlying signaling mechanisms.

## Materials and Methods

### Cell Culture and Stimulation

Cell culture and differentiation of THP-1 cells into macrophages were described previously (Dai et al., 2017a). The THP-1 cell line was obtained from the Cell Bank of the Chinese Academy of Sciences (Shanghai, China) and incubated in RPMI media supplemented with 10% FBS and 1% penicillin–streptomycin. The cells were induced to differentiate into macrophages by 300 ng/ml phorbol 12-myristate 13-acetate for 72 h (PMA, Sigma-Aldrich, Darmstadt, Germany). Twenty-four hours later, the cells were used in the experiments.

For stimulation, the cells were incubated with 15 mM (HG1), 30 mM (HG2), or 45 mM (HG3) glucose for 12 h. In osmotic pressure control (OP) group, we added 30 mM mannitol to exclude a hyperosmolar effect.

For treatment, rapamycin (10 nM–200 nM, Abcam, Cambridge, UK), BAY 11-7082 (10 μM, Abcam, Cambridge, UK), and pyrrolidine dithiocarbamate (PDTC) (5 μM, Abcam, Cambridge, UK) were added to the cultures simultaneously with a high glucose priming step and were maintained until assays were performed.

### Western Blot Analysis

Cell lysates were resolved by SDS-polyacrylamide gel electrophoresis and transferred to polyvinylidene difluoride (PVDF) membranes. The membranes were blocked with 5% skim milk in Tris-buffered saline for 2 h at room temperature and then incubated with primary antibodies against NLRP3, Caspase-1, ASC, mTOR, p-mTOR, P65, p-P65, P70s6k, p-P70s6k, IκBα, or β-actin overnight at 4°C. The membranes were subsequently incubated with secondary antibodies. After washing with TBST, the bands were detected by chemiluminescence (ECL) and imaged with X-ray films. β-actin was used as an endogenous reference for normalization. Phosphorylation sites included p-mTOR(Ser2448), p-P65 (Ser536), and p-P70s6k (Ser424).

### Cell Viability Analysis

THP-1-derived macrophages were seeded into 96-well plates at a density of 1×10^5^ cells per well with RPMI 1640 medium containing 10% FBS with high glucose (30 mM) or mannitol (30 mM) or rapamycin (50 nM). The cells were cultured under normal conditions for 72 h, and cell viability was detected by using a cell counting kit-8 (CCK-8) assay at 24, 48, and 72 h. Briefly, 10 µl of CCK-8 solution (CK04, Dojindo, Japan) was added, and then the cells were cultured under normal conditions for an additional 4 h. Finally, the absorbance at 490 nm was measured.

### Transient Transfection

Each group of samples was collected with 6×10^5^ THP-1 cells to 15 ml centrifuge tubes. The cells were resuspended in 2 ml serum-free MEM medium and centrifuged at 1,000 g for 2 min. The cells was collected and resuspended in 100 μl opti-MEM. On the other hand, 50 ul opti-MEM was added to two 1.5 ml centrifuge tubes, and then 10 ul Lipofectmaine 2,000 and 120 ul siRNA (double-stranded annealed before FITC labeling) were added respectively and mixed well. After 10 min at room temperature, two dilution products were mixed and added to the above 100 ul cell suspension. After transferred in a water bath at 37°C for 30 min, the cells were transferred to a 12-well culture plate (pre-add 500 ul of RPMI1640 medium containing 2% FBS), and incubated overnight at 37°C and 5% CO_2_. The cells were centrifuged at 1,000 g for 2 min and added with 2 ml RPMI1640 medium containing 10% FBS, and then transferred to a 6-well culture plate. Cells were cultured for 24 h under normal conditions. The cell transfection efficiency was observed by fluorescence microscopy. The cell transfection efficiency = the number of cells with FITC expression/the total number of cells in the field of view. Five groups of fields were randomly selected from each group for observation.

siRNA- mTOR (NM_004958.3): 5′-GAAGAACTGCGTCATGCC-3′; NC:5′-GAAGCCAGATCCAGCTTCC-3′; siRNA-P65(NM_021975.4): 5′-GATCTGCCGAGTGAACCGA-3′; NC:5′-GAAGCCAGATCCAGCTTCC-3′.

### Immunofluorescent Staining

For the NF-κB translocation assay, THP-1-derived macrophages were induced with high glucose in the presence or absence of rapamycin. The cells were fixed with 95% acetone for 30 min and washed with PBS for an additional 5 min. Immunostaining was performed using rabbit anti-p65 antibody (1:350 dilution), followed by Alexa Fluor 488-conjugated goat anti-mouse IgG antibody (1:3,000 dilution, Abcam, Cambridge, UK). After washing, the cells were stained with 20 μL Hoechst 33258 (Invitrogen, USA) for 3 min. Then, slides were washed and mounted with glycerol jelly mounting medium and photographed with a fluorescence microscope.

### Measurement of Relative mRNA Expression by Real-Time PCR

THP-1-derived macrophages were collected by centrifugation 24 h after transfection, washed with pre-cooled dPBS, supplemented with pre-cooled Trizol reagent, and pipetted until the lysate became viscous and clear. The lysate was transferred to a 1.5 ml centrifuge tube and extracted using the phenol–chloroform method. The purity of the total RNA was determined by ultraviolet spectrophotometry, and the mRNA levels of P65 and mTOR were detected by real-time PCR. Total RNA (2 μg) was used as the template to prepare cDNA by reverse transcription. The product of reverse transcription (2 μl) was used as the template for real-time PCR. The results were analyzed using the 2^ΔΔCt^ method with β-actin as the internal reference. The PCR primers were as follows: β actin(NM_001101.5)-forward, 5′-CTCCCCCATGCCATCCTGCGTCTG-3′, β actin-reverse, 5′-CTCGGCCGTGGTGGTGAAGC-3′; P65(NM_021975.4)-forward, 5′-CTCCGCGGGCAGCATCC-3′; P65-reverse, 5′-CATCCCGGCAGTCCTTTCCTACAA-3; mTOR(NM_004958.4)-forward, 5′-GTAGCCGCCCTTCGTGCCTGTCT-3′;mTOR-reverse, 5′-ATCCCGATTCATGCCCTTCTCTTT-3. The experiment was carried out on a Takara fluorescent quantitative TP800.

### Statistical Analysis

Experiments were independently repeated at least three times, with at least three samples per group in each experiment. SPSS 18.0 statistical software (SPSS Inc, Chicago, USA) was used for data analysis. Data are shown as the mean ± SD. Statistical analysis was performed using Student’s t-test or one-way ANOVA followed by Tukey’s *post hoc* tests. P values less than 0.05 indicated statistical significance.

## Results

### Rapamycin Reduces NLRP3 Inflammasome Activation in Macrophages

**Figure 1:**
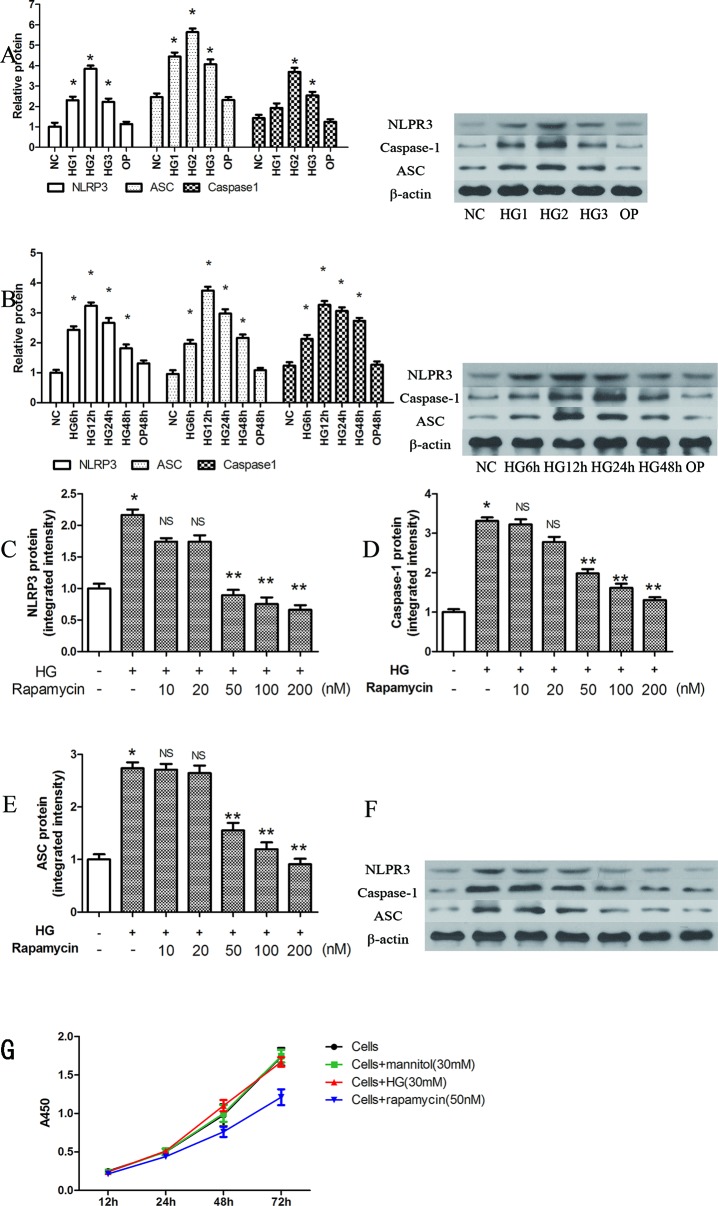
Rapamycin reduces NLRP3 inflammasome activation in macrophages. **(A**, **B)** The expression of NLRP3, Caspase-1 and ASC protein assessed by western blot after high glucose treatment by concentration and time course. *p 0.05 versus NC group. Data are expressed as mean ± SD, n = 3. **(C**–**F)** Western blot indicated that rapamycin dose-dependently reduced the expression of NLRP3, Caspase-1 and ASC protein. **(G)** Cells were treated with high glucose (30mM) or mannitol (30mM) or rapamycin (50nM), and cell viability were measured using the CCK-8 assay. *p 0.05 versus control; **p 0.05 versus HG group; NS, not significant compared with HG group. Data are expressed as mean ± SD, n = 3.

NLRP3, ASC and caspase-1 were significantly induced by several high concentrations of glucose at 12 h, when compared with that of the control group (P < 0.05). The highest relative protein expression was found in the 30 mM high glucose group (**Figure 1A**).

NLRP3, ASC and caspase-1 were also significantly induced after 6, 12, 24, and 48 h of exposure to 30 mM glucose (**Figure 1B**, P < 0.05). We observed the highest levels of protein at 12 h (**Figure 1B**). In the OP group, mannitol had no significant effect on protein expression (P > 0.05).

After pretreatment with different concentrations of rapamycin for 2 h, NLRP3, ASC and caspase-1 were decreased. The protein levels were significantly reduced when the concentration of rapamycin was greater than 50 nM (P < 0.05). Rapamycin reduced the expression of NLRP3, ASC and caspase-1 in a concentration-dependent manner (**Figures 1C–F**).

We also evaluated cell viability with the CCK-8 assay to determine the effects of high glucose (30mM) or mannitol (30 mM) or rapamycin (50 nM) on cells (**Figure 1G**). The results showed that high glucose (30 mM) and mannitol (30 mM) did not significantly alter cell viability. Treatment with 50 nM rapamycin significantly decreased the proliferation of cells after 24 h treatment. In our study, the cells were pretreated with rapamycin for 2 h.

### Rapamycin Reduces NLRP3 Inflammasome Activation by Inhibiting Mtor Phosphorylation

**Figure 2:**
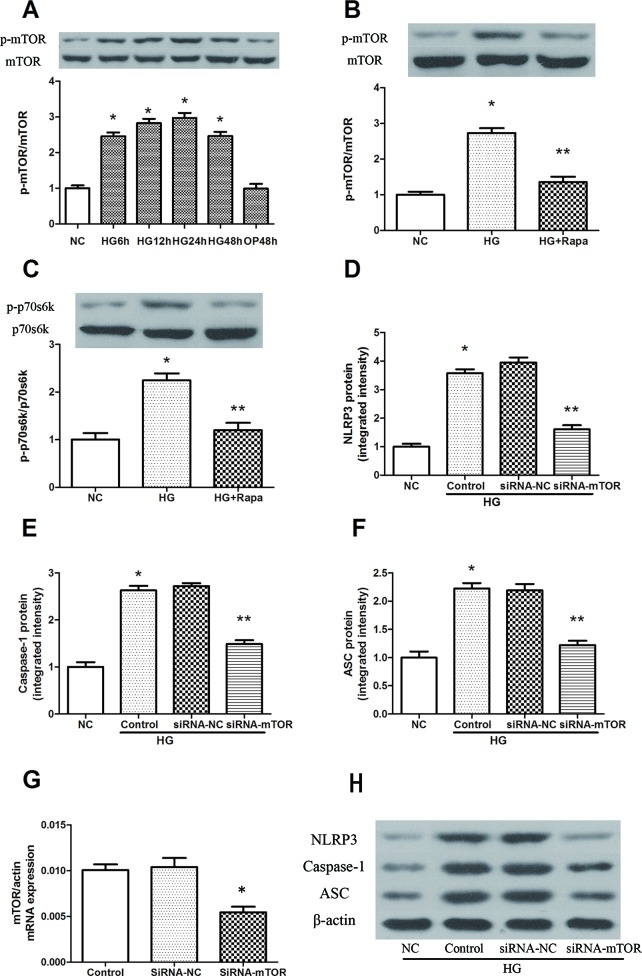
Rapamycin reduces NLRP3 inflammasome activation by inhibiting mTOR phosphorylation. **(A)** The expression of p-mTOR/mTOR assessed by western blot after high glucose treatment by time course. **(B**, **C)** mTOR and p70s6k phosphorylation analyzed by western blot after rapamycin treatment. **(D**–**F**, **H)** Western blot indicated that knockdown of mTOR significantly reduced the expression of NLRP3, Caspase-1 and ASC protein. *p 0.05 versus NC group; **p 0.05 versus HG or HG (control) group. Data are expressed as mean ± SD, n = 3. **(G)** Real-time PCR indicated that Knocking down mTOR significantly reduced the mRNA expression of mTOR. *p 0.05 versus siRNA NC group. Data are expressed as mean ± SD, n = 3.

High glucose induced an increase in mTOR phosphorylation in a time-dependent manner, and the maximal effect was observed at 24 h following 30 mM glucose treatment (**Figure 2A**). As an inhibitor of mTOR, rapamycin significantly inhibited the phosphorylation of mTOR, while the phosphorylation level of mTOR was effectively increased in macrophages after high glucose treatment (**Figure 2B**). The 70-kDa ribosomal protein S6 kinase (P70s6k) is a downstream protein of mTOR, and was significantly increased 12 h after 30 mM glucose treatment, while rapamycin pretreatment effectively reduced the phosphorylation of P70s6k (**Figure 2C**). In addition, mTOR knockdown significantly reduced the protein expression of NLRP3, ASC and caspase-1 in macrophages (**Figures 2D–H**). These findings suggest that rapamycin decreases NLRP3 inflammasome activation by inhibiting mTOR phosphorylation.

### Rapamycin Reduces NLRP3 Inflammasome Activation by Inhibiting NF-κb Activation

**Figure 3:**
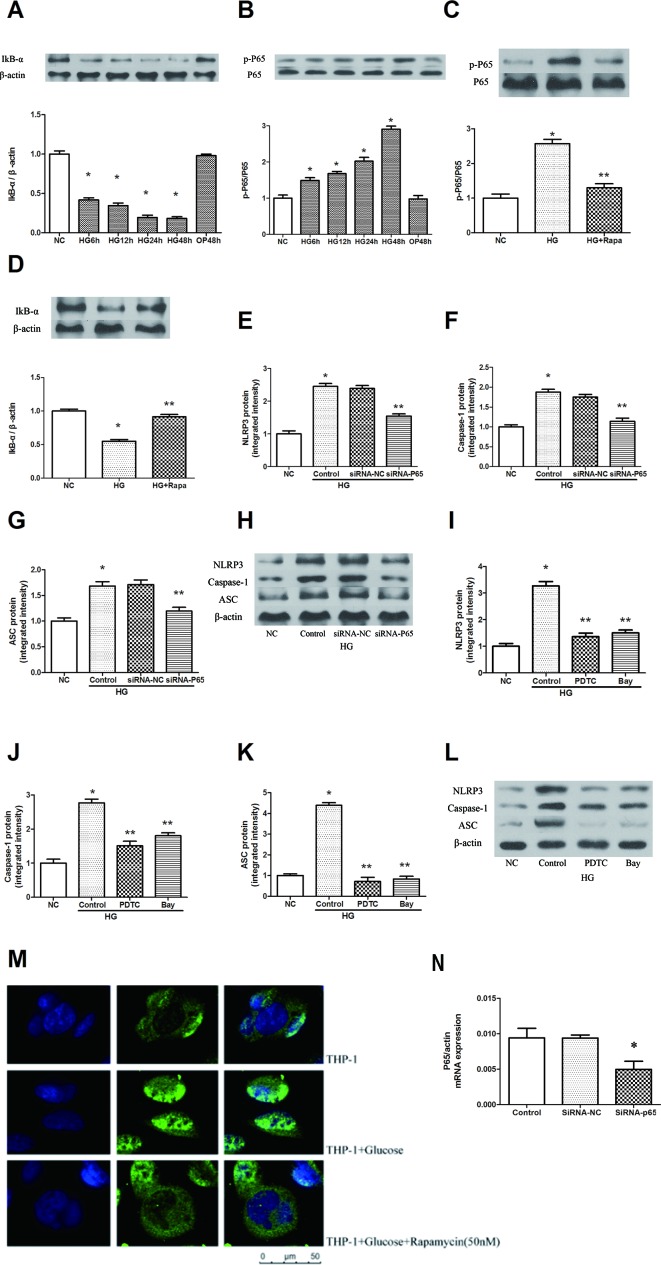
Rapamycin reduces NLRP3 inflammasome activation by inhibiting NF-κB activation. **(A**, **B)** The expression of p-P65/P65 and IκBα assessed by western blot after high glucose treatment by time course. **(C**, **D)** P65 phosphorylation and IκBα analyzed by western blot after rapamycin treatment. **(E**–**H)** Western blot indicated that knockdown of P65 significantly reduced the expression of NLRP3, Caspase-1 and ASC protein. **(I**–**L)** Western blot indicated that treatment with NF-κB inhibitors significantly reduced the expression of NLRP3, Caspase-1 and ASC protein. **(M)** Translocation of NF-κB p65 was observed after high glucose treatment for 12h under a fluorescence microscope. *p 0.05 versus NC group; **p 0.05 versus HG or HG (control) group. Data are expressed as mean ± SD, n = 3. **(N)** Real-time PCR indicated that Knocking down p65 significantly reduced the mRNA expression of p65. *p 0.05 versus siRNA NC group. Data are expressed as mean ± SD, n = 3.

High glucose increased the phosphorylation of P65 in a time-dependent manner, and the maximal effect was observed at 48 h following 30 mM glucose treatment (**Figure 3B**). The inhibitor of NF-κB signaling, IκBα, was effectively decreased after high glucose treatment in a time-dependent manner (**Figure 3A**, P < 0.05). Pretreatment with 50 nM rapamycin for 2 h inhibited the high glucose induced IκBα degradation and p-P65 expression in macrophages (**Figures 3C, D**). Immunofluorescence staining showed that p65 was predominantly localized in the cytoplasm in macrophages. A significant accumulation of p65 was found in the nucleus following high glucose treatment. This result indicates that high glucose induces the redistribution of p65 from the cytoplasm to the nucleus, whereas this effect was attenuated by rapamycin (**Figure 3M**). In addition, we inhibited the expression of NF-κB by NF-κB knockdown and the NF-κB inhibitors Bay 11-7082 and PDTC (**Figure S3**). The protein expression levels of NLRP3, ASC and caspase-1 decreased compared with those of the HG group in macrophages (**Figures 3E–L, N**, P < 0.05). These findings suggest that rapamycin reduces NLRP3 inflammation activation by inhibiting NF-κB activation.

### mTOR siRNA Suppresses NF-κb Activation, Resulting in the Inhibition of NLRP3 Inflammasome Activation

**Figure 4:**
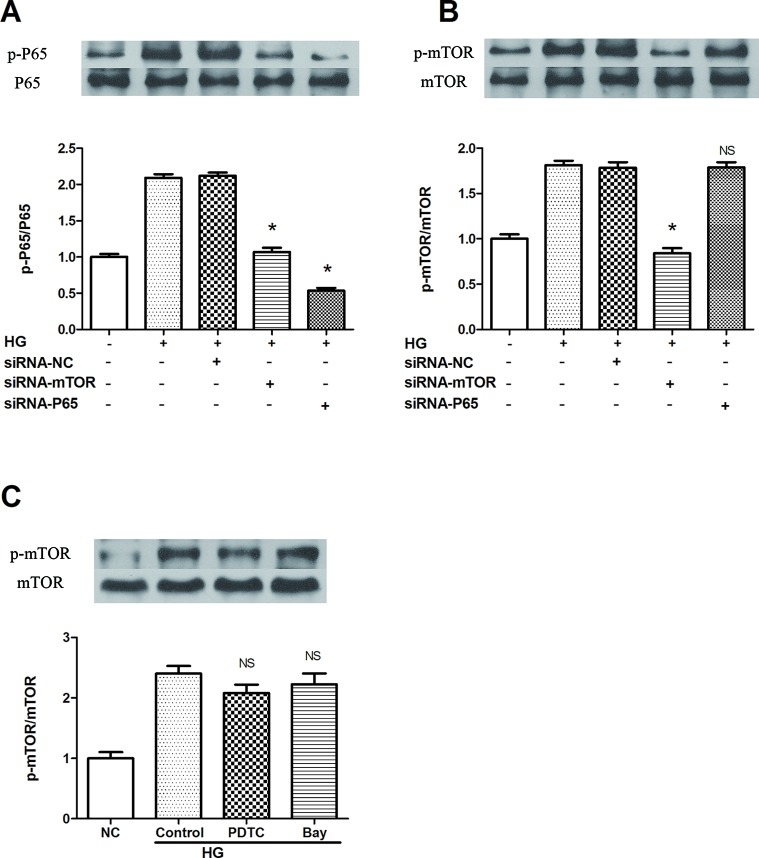
mTOR siRNA inhibits NF-κB activation, resulting in the suppression of NLRP3 inflammasome activation. **(A**, **B)** P65 and mTOR phosphorylation analyzed by western blot after mTOR and P65 knockdown. **(C)** mTOR phosphorylation analyzed by western blot after NF-κB inhibitors treatment. *p 0.05 versus HG group; NS, not significant compared with HG or HG (control) group. Data are expressed as mean ± SD, n = 3.

To clarify the specific signaling mechanism by which rapamycin reduces NLRP3 inflammasome activation in macrophages, upstream and downstream relationships were assessed. Both mTOR and NF-κB knockdown decreased the phosphorylation of p65 in cells treated by 30 mM glucose for 12 h (**Figures 4A, B**). On the other hand, NF-κB knockdown and NF-κB inhibitors (BAY 11-7082 and PDTC) did not inhibit mTOR phosphorylation 12 h after 30 mM glucose treatment (**Figure 4C**). These outcomes suggest that mTOR knockdown leads to the suppression of downstream activation of NF-κB, followed by the inhibition of NLRP3 inflammasome activation.

## Discussion

Previous research has indicated that NLRP3 inflammasome activation is one of the key contributors to impaired wound healing in diabetes(Bitto et al., 2014). In this study, high glucose increased the activity of mTOR and NF-κB and induced NLRP3 inflammasome expression in macrophages. We also showed that rapamycin could ameliorate high glucose-induced NLRP3 inflammasome activation by inhibiting the mTOR/NF-κB signaling pathway in macrophages.

Chronic wounds are normally associated with a persistent inflammatory response. The NLRP3 inflammasome has been demonstrated to play a crucial role in the chronic inflammatory response (Mirza et al., 2014). Our previous study reported high expression of the NLRP3 inflammasome and IL-1β in macrophages isolated from human diabetic wounds and in high glucose-induced macrophages (**Figure S1**) (Zhang et al., 2017). Therefore, inhibiting inflammasome activation *in vivo* with topical application of pharmacological inhibitors might offer a new target for improving wound healing in diabetic individuals.

Rapamycin, an mTOR inhibitor, is a potent immunosuppressive drug that is widely applied after renal, liver, and heart transplantation (Jia et al., 2019). Xiao et al. (2013) reported that rapamycin promoted wound healing by enhancing autophagy in the epidermis in a rat model of deep second-degree burn wounds. However, it was also reported that rapamycin delayed wound healing *via* a γδ T cell defect in skin tissue (Mills et al., 2008). Given these multiple and contradictory roles of the mTOR pathway, we hypothesized that differential doses of rapamycin might lead to different consequences in wound healing. Previous studies have shown that mTOR actively suppresses autophagy, which is one of the predominant mechanisms that mediates NLRP3 activation and IL-1β secretion (Jung et al., 2009; Dai et al., 2017a). In this study, we showed that high glucose upregulates the expression of mTOR phosphorylation in macrophages and that suppressing mTOR phosphorylation using rapamycin or mTOR siRNAs causes a decrease in NLRP3 inflammasome activation. These outcomes suggest that rapamycin might attenuate NLRP3 inflammasome activation in macrophages by inhibiting mTOR phosphorylation.

NF-κB is the most important nuclear transcription factor that regulates various cellular processes in the inflammatory response (Zhang et al., 2015). Increasing evidence indicates that rapamycin shows anti-inflammatory actions *via* regulating NF-κB activity (Liu et al., 2017). Our data suggested that suppression of NF-κB activation by rapamycin led to a decrease in high glucose-induced NLRP3 inflammasome activation. High glucose upregulates NADPH oxidase and ROS generation, causing activation of redox-sensitive NF-κB. Subsequently, activated NF-κB promotes NLRP3 inflammasome activation, which in turn mediates IL-1β and IL-18 production and contributes to the persistent inflammatory response (Zhou et al., 2016).

The inhibition of NF-κB by rapamycin may be explained by the fact that mTOR activates the endogenous inhibitor of NF-κB kinase (IKK) (Han et al., 2008). mTOR mediated induction of IKK leads to the activation of NF-κB (Ahmad et al., 2013). NF-κB is an inactive heterodimer composed of p50 and p65 subunits interacting with a member of the inhibitory IκB family in resting cells (Bai et al., 2016). Upon stimulation by activating signals, the IKK complex phosphorylates IκBα, promoting its degradation and freeing NFκB p50/p65 to translocate to the nucleus and initiate transcription (Wang et al., 2017; Morgan et al., 2019). In our study, mTOR deficiency suppressed NF-κB activation followed by decreased levels of the NLRP3 inflammasome. Therefore, these results indicate that mTOR is upstream of NF-κB and that activated NF-κB is induced by high glucose.

For suspension cells, low transient transfection efficiency is a key factor affecting the effect of gene intervention. In this study, we used Lipofectmaine 2000 transfection reagent and an optimized transfection protocol to allow siRNA to achieve a 50% transfection efficiency in THP-1 cells (48 h after transfection) (**Figure S2**). In addition to adjusting the amount of siRNA and liposome, we mainly reduced the cell culture system, and controlled the proliferation rate of the transfected cells by reducing the serum content. From these two aspects, we have effectively increased the time and probability of inoculation of liposome and SiRNA complexes with cell membrane, which may be the primary cause for achieving ideal transfection efficiency.

In this study, we focused on the roles of rapamycin in the suppression of high glucose-induced NLRP3 inflammation activation. After high glucose treatment, the inflammatory reaction in macrophages was inhibited by rapamycin. Therefore, rapamycin might act as a possible therapeutic option for high glucose-induced inflammatory response in impaired wound healing in the future. An *in vivo* study to assess the clinical relevance of our findings would help confirm the implication of inflammasome modulation for the treatment of disease.

## Data Availability Statement

The raw data supporting the conclusions of this manuscript will be made available by the authors, without undue reservation, to any qualified researcher.

## Author Contributions

Conceptualization: HC. Investigation: JD. Methodology: CJ. Writing – original draft: JD. Writing – review and editing: HC and YC.

## Funding

This work was supported by National Natural Science Foundation of China 81702140 and the Interdisciplinary Program of Shanghai Jiao Tong University (YG2017QN18).

## Conflict of Interest

The authors declare that the research was conducted in the absence of any commercial or financial relationships that could be construed as a potential conflict of interest.

## Supplementary Material

Figure S1 NLRP3 inflammasome expression in THP-1 cells (n = 3) and macrophages derived from diabetic wounds (n = 6).

Figure S2 Forty-eight hours after transfection (si RNA-NC).

Figure S3 Inhibition rate of P65.
