# Supplementary figures and images for: Rapamycin Attenuates High Glucose-Induced Inflammation Through Modulation of mTOR/NF-κB Pathways in Macrophages
Source: Front Pharmacol. 2019 Oct 30;10:1292. doi: 10.3389/fphar.2019.01292 (PMC6831745; doi:10.3389/fphar.2019.01292)

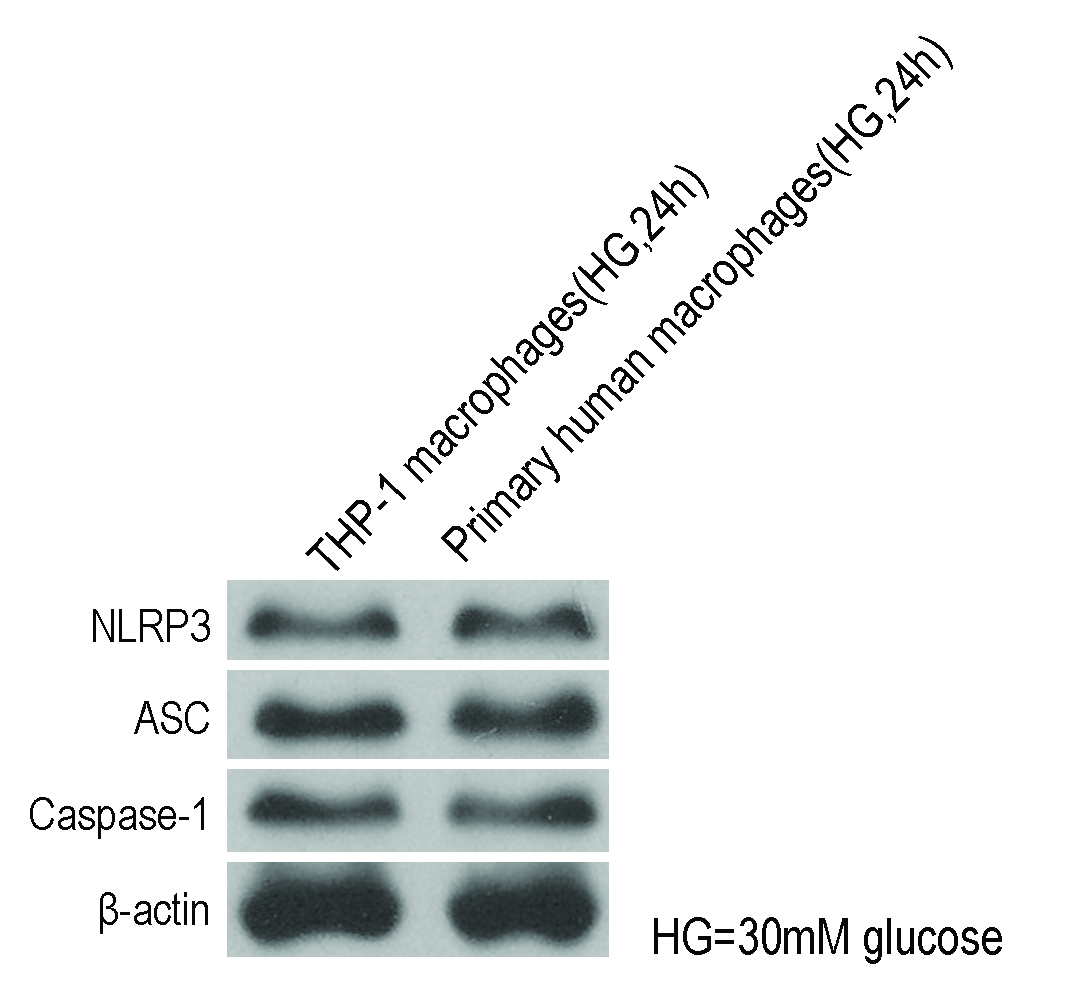

Supplement: Figure S1 — NLRP3 inflammasome expression in THP-1 cells (n = 3) and macrophages derived from diabetic wounds (n = 6). [file Image_1.tif]

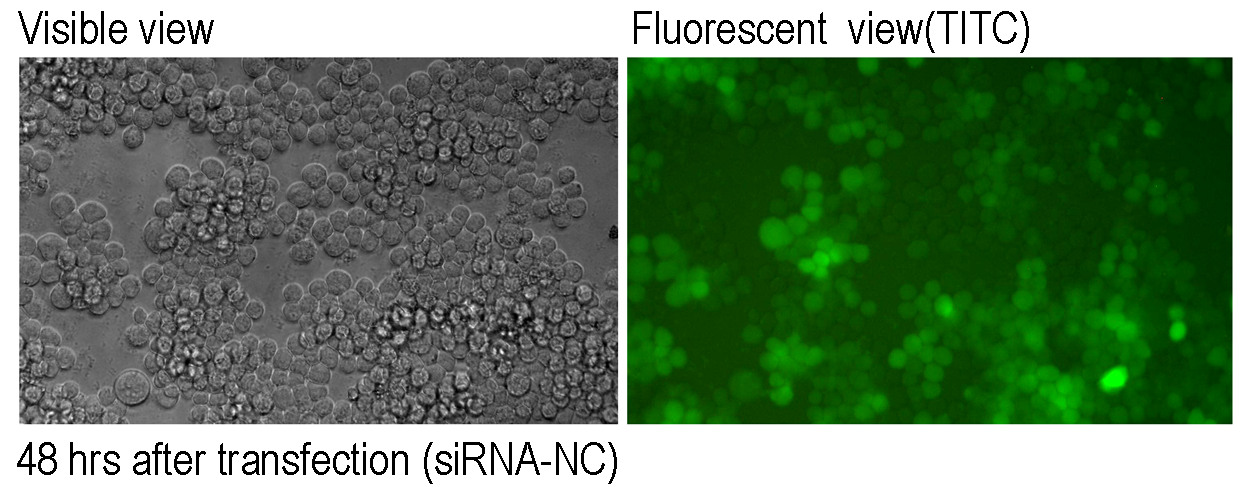

Supplement: Figure S2 — Forty-eight hours after transfection (si RNA-NC). [file Image_2.tif]

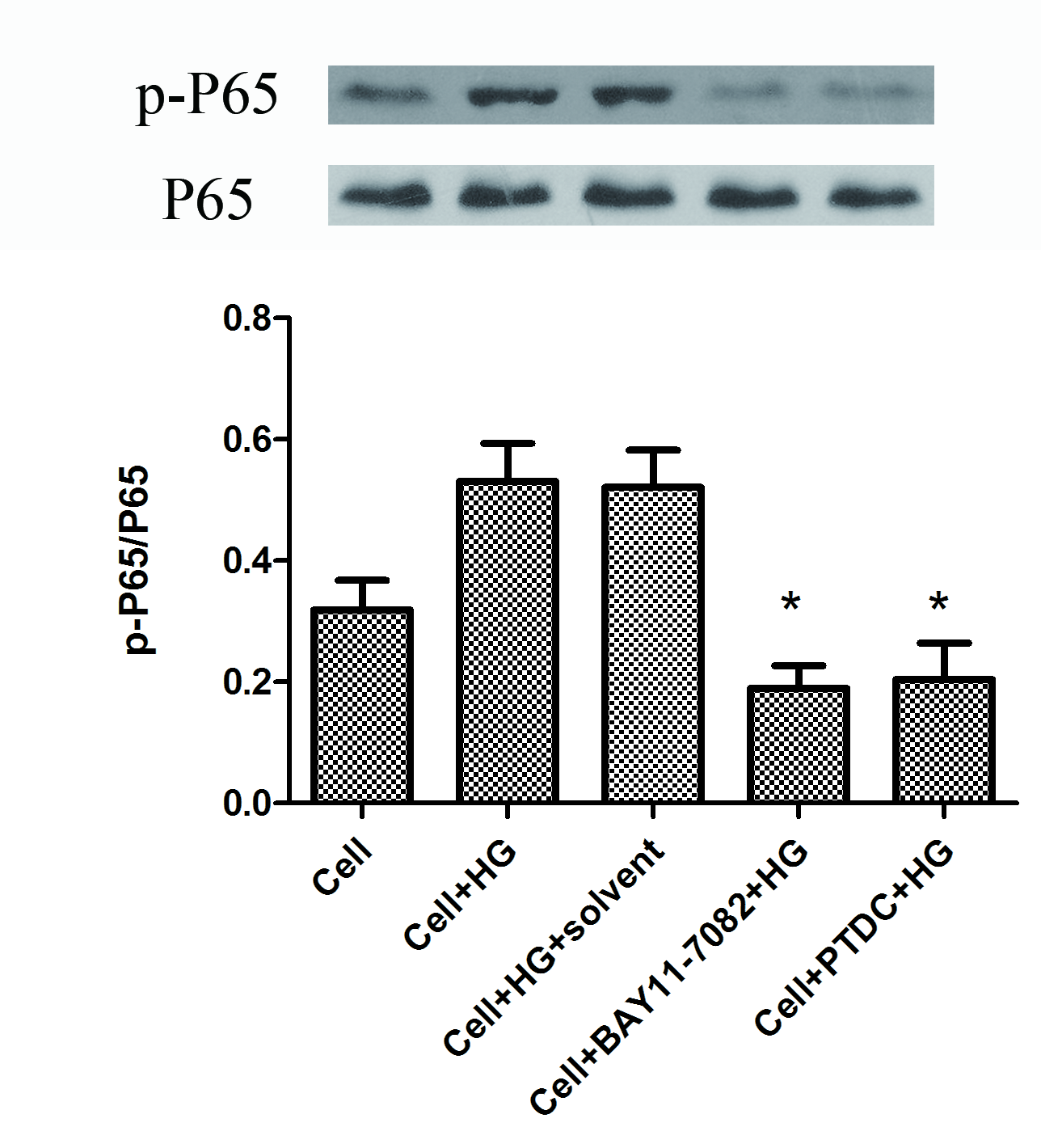

Supplement: Figure S3 — Inhibition rate of P65. [file Image_3.tif]
